# The Folded Paper Size Illusion: Evidence of Inability to Perceptually Integrate More Than One Geometrical Dimension

**DOI:** 10.1177/2041669516658048

**Published:** 2016-07-07

**Authors:** Claus-Christian Carbon

**Affiliations:** Department of General Psychology and Methodology, University of Bamberg, Bamberg, Germany Research group EPÆG (Ergonomics, Psychological Æsthetics, Gestalt), Bamberg, Germany Bamberg Graduate School of Affective and Cognitive Sciences (BaGrACS), Bamberg, Germany

**Keywords:** art and illusion, perception, didactics, insight, visual processing, area estimation, alignment, folded paper size-illusion, psychophysics, geometrical fractional algorithm

## Abstract

The folded paper-size illusion is as easy to demonstrate as it is powerful in generating insights into perceptual processing: First take two A4 sheets of paper, one original sized, another halved by folding, then compare them in terms of area size by centering the halved sheet on the center of the original one! We perceive the larger sheet as far less than double (i.e., 100%) the size of the small one, typically only being about two thirds larger—this illusion is preserved by rotating the inner sheet and even by aligning it to one or two sides, but is dissolved by aligning both sheets to three sides, here documented by 88 participants’ data. A potential explanation might be the general incapability of accurately comparing more than one geometrical dimension at once—in everyday life, we solve this perceptual-cognitive bottleneck by reducing the complexity of such a task via aligning parts with same lengths.

Visual illusions are fun, but they are also insightful (Carbon, 2014)—the great pedagogic value behind such illusions is that most readers, while being amused, also experience perceptual insights which assist the understanding of rather complex perceptual processing (see Gregory, 2009). Here, I present a very simple illusion which was inspired by a discussion which I involuntarily witnessed in a German photographic shop back in the year 1989, when photoprints in Germany were typically made in sizes of 9 × 13 cm (*small purchase option*) or 13 × 18 cm (*large option*). The customer who was currently being served was complaining about the incomprehensible billing policy of the shop—from his perspective, the large purchase option was only marginally larger than the small option, but incomparably more expensive. Indeed, when we looked at the specific setting, with the smaller sized print being centered on the larger one, the larger photo print looked only marginally larger. Ignoring all mathematically based means, the vendor followed this perceptual path, puzzling about the pricing policy himself. After a while, I chimed into the conversation with a simple mathematical argument: to compare the size of a 9 × 13 cm and a 13 × 18 cm print, just “cancel the common 13” and you will get a doubled remaining side as 18 = 2 × 9, so the larger option is 100% larger than the smaller one, the size is doubled! Both attendees looked through me, fully confused, a bit shocked and very doubtful . . . doubled? No way! They did not find basic mathematical rules convincing at all. So I decided to change my persuasion strategy (purely for practical reasons; I was late and wanted to be served quickly!) by *visually* demonstrating the fractional arithmetic via a very subtle, but extremely insightful change of the configuration: simply by rotating the smaller print by 90° and positioning it in such a way that the large edge was aligned with the small edge of the large print, both attendees exclaimed “wow!” in unison. Instantly, they had understood the size relationship of the two prints—the larger print was really double the small one; exactly double!

Twenty-five years later, we can easily replicate the whole setting, simulating this aha!-insight effect (inspect therefore particularly Figure 1(a) and (g)): Just take two sheets of paper (e.g., A4); one original-sized, one halved by folding, and compare them in terms of area size by centering the halved sheet on the center of the original one! We perceive the larger sheet as far less than double the size of the smaller one. For instance, by asking people to assess how much bigger the larger sheet is compared with the smaller one (in percentage; thus, “100%” would be the correct answer^1^); most people strongly underestimate the size of the larger sheet. When I asked 102 participants (undergraduates of psychology, 76 female, mean age 20.8 years), two main results were retrieved: (a) although the entrance requirements for starting the study of psychology are extremely high in Germany, 14 persons were not able to operate with percentages in a meaningful way, an inability which is not at all uncommon (Siegler & Lortie-Forgues, 2015) besides severe forms of problems with numbers and problems applying mathematical routines such as dyscalculia (with a prevalence rate of about 6%, see Shalev, Manor, Auerbach, & Gross-Tsur, 1998)—consequently these persons were excluded from further analyses; and (b) remaining participants (88 undergraduates of psychology, 68 female, mean age 20.9 years) showed the same aha-effect as was described in the photographic shop after having had rotated the sheets. Before doing this, I confronted them with a series of geometrical settings (see the top row of Figure 1 for the A4 settings), first consisting of an A4 and a halved A4 (i.e., A5) sheet, always starting with the setting shown on the very left side, progressing to the version on the very right side, one after another. After this A4 series, the same general settings were shown for the U.S.-letter size plus the halved U.S. letter (“half letter”), see the bottom row of Figure 1. Participants assessed the configurations of the first “A4” series very similarly to the second “letter” series—exact values plus effect sizes for one-sample *t*-tests against the true value of 100% can be retrieved from Figure 1.

**Figure 1.**
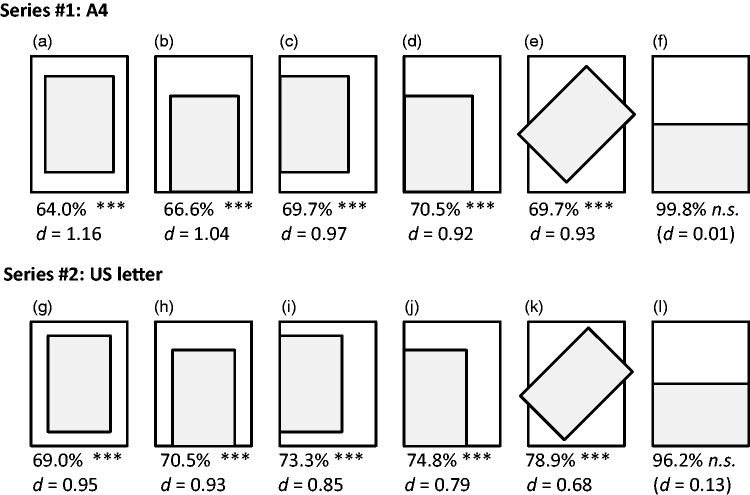
Overview of the employed experimental conditions, already in the experimental order which was realized, starting with a centered version (a/g) and always ending with a fully aligned version (f/h)—the participants were first exposed to the A4 paper size setting (Series #1), then to the U.S. letter size setting (Series #2). Percentage values show the mean estimations of how much bigger the larger sheet is compared with the smaller one (100% would be the correct answer, e.g., 64.0% in the case of Figure 1(a) means that the area of the bigger sheet is strongly underestimated, *d* = 1.16); *** indicate *p-*values < .001. Effect sizes are expressed as (Cohen’s) *d*’s for one-sample *t*-tests against 100%.

The main result was that for all conditions but the last one of both series (Figure 1(f) and (l)), we obtained medium-to-large effects (Cohen’s *d* ’s > 0.68; for A4-settings always *d* ’s > 0.93 indicating large effects through-out) in terms of deviations from the true value of 100%, meaning that the area size of the large rectangle was strongly underestimated. This effect was strongest when the small rectangle was centered to the large one (Cohen’s *d* ’s > 0.95)—and even rotating and aligning it to one single side did not dismiss this very large perceptual effect. The only way of escaping this strong visual illusion was to align two sides of both sheets at once in such a way that the small rectangle halved the large one (Figure 1(f) and (l)). This provides some indication of how the effect emerges: We seem to face a general incapability of accurately comparing more than one geometrical dimension at once. Such effects are already known from other illusions and perceptual phenomena. For instance, Piaget, Inhelder, and Szeminska (1960) already showed that (primary school) children predominantly used just one single dimension (height) to estimate volume, the so-called *centration hypothesis*—but see counter-evidence in later work where the integration of all three dimensions have been documented (Ebersbach, 2009). That adults are also susceptible to such volume illusions has been extensively documented, even for everyday-life objects such as typical food and drink packages (Raghubir & Krishna, 1999)—again, it seems that simple measures or single dimensions are predominantly used to estimate more complex measures or to predict the outcomes of actions by analyzing two concurrent movements (Hergovich, Grobl, & Carbon, 2011). Perceptual research, however, also offers some alternative explanations for the revealed effect. First of all, the general tendency to underestimate areas documented by psychophysical power functions with exponents below one (e.g., Ekman & Junge, 1961) cannot account for the effect seen here as the relationships between the two physical areas remained constant at 2:1 across all conditions. Assimilation theory (Pressey, 1967, 1971) appears to offer a more promising account. According to this theory, in a group of objects the ones with the extreme values, for example, lengths, will be misperceived toward the average values (the theory has many names in science, for example, “regression to the mean” in statistics or “central tendency error” in applied fields). In the given case, people would adapt their length or area assessments toward the mean of the given context lines or areas, respectively, so would underestimate the size of the bigger rectangular area. However, assimilation theory will be quite ambiguous in predicting the specific outcome for the cases of 45° rotated configurations depicted in Figure 1(e) and (k), as it is not clear how the edges will assimilate—with the smaller or longer ones? Another alternative explanation arises by comparing the area of the smaller object and the non-covered, residual of the larger rectangle. As is seen particularly clearly in the centered cases in Figure 1(a) and (g), the residual area is quite narrow, in fact much narrower than the smaller object. Indeed, we observe particularly large effects in Figure 1(a) and (b) as well as in Figure 1(g) and (h). Further qualification of such an explanation, however, reveals that the resulting underestimation of the summed up narrow areas is again an indication of problems in integrating different dimensions; we could also reconcile it with assimilation theory operating with areas instead of lengths.

In everyday life, we solve such perceptual-cognitive bottlenecks by reducing the complexity of such a task via aligning parts with same lengths; actually, what Figure 1(f) and (l) provides is a kind of geometrical fractional arithmetic as one side of the first object fully cancels out one side of the second—to be compared—object. This reduces the degrees of freedom to just one—now only one remaining side has to be compared with the other side, and this works out brilliantly as shown by data provided in Figure 1 (for versions 1(f) and (l)). If we follow such a strategy to reduce the complexity of this perceptual task, we are able to validly estimate “area sizes”—in fact we are then only estimating unidimensional information—if we do not follow such a strategy, we evidently and substantially fail. The most important insight from the entire story seems to be: sometimes, insights from perceptual illusions are much more striking than even mathematical proof, particularly if such illusions show a high degree of *Prägnanz*! Finally, the enjoyment of perceiving such an emerging Gestalt and the insight it creates gives us the pleasure needed to continue in our path of perceptual learning (Muth & Carbon, 2013).

## Note

1 In fact and wisely remarked by a reviewer, the metric might be suboptimal, mainly because we usually think of percentages as parts of something bigger but the reverse less often. Nevertheless, the effect seems to be extremely robust across viewing conditions, instructions, and types of measurement as the typical beholder of this illusion, plus a wide range of people attending public talks when I demonstrated it and utilized different metrics, could authentically report.
